# *N*′-[(*E*)-(3-Phenyl-1*H*-pyrazol-4-yl)methyl­idene]naphtho­[2,1-*b*]furan-2-carbohydrazide monohydrate

**DOI:** 10.1107/S1600536811050239

**Published:** 2011-11-30

**Authors:** Hoong-Kun Fun, Wan-Sin Loh, Shridhar Malladi, B. M. Ganesh, Arun M. Isloor

**Affiliations:** a X-ray Crystallography Unit, School of Physics, Universiti Sains Malaysia, 11800 USM, Penang, Malaysia; b Medicinal Chemistry Division, Department of Chemistry, National Institute of Technology-Karnataka, Surathkal, Mangalore 575 025, India

## Abstract

In the title hydrate, C_23_H_16_N_4_O_2_·H_2_O, the pyrazole ring is approximately planar, with a maximum deviation of 0.023 (1) Å, and makes dihedral angles of 28.63 (6) and 46.44 (7)° with the naphtho­[2,1-*b*]furan ring system and the benzene ring, respectively, In the crystal, O—H⋯N, O—H⋯O, N—H⋯O, N—H⋯N, C—H⋯O and C—H⋯N hydrogen bonds link the mol­ecules, forming sheets lying parallel to the *ab* plane. The crystal structure also features C—H⋯π inter­actions involving the centroids of the pyrazole and benzene rings.

## Related literature

For the biological activity of hydrazides, hydrazones and their adducts, see: Jahagirdar *et al.* (1990 ▶); Cavier & Rips (1965 ▶); Silva *et al.* (2005 ▶); Eissa & Soliman (2009 ▶). For a related structure, see: Choi *et al.* (2009 ▶). For the stability of the temperature controller used in the the data collection, see: Cosier & Glazer (1986 ▶).
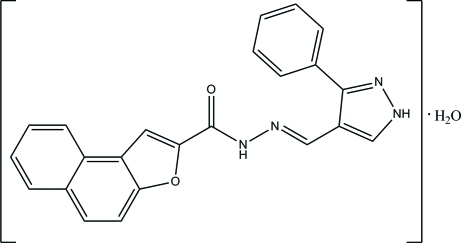


## Experimental

### 

#### Crystal data

C_23_H_16_N_4_O_2_·H_2_O
*M* *_r_* = 398.41
Monoclinic, 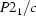

*a* = 7.1383 (1) Å
*b* = 9.3928 (1) Å
*c* = 28.4200 (4) Å
β = 96.864 (1)°
*V* = 1891.86 (4) Å^3^
*Z* = 4
Mo *K*α radiation
μ = 0.10 mm^−1^
*T* = 100 K
0.31 × 0.25 × 0.18 mm

#### Data collection

Bruker SMART APEXII CCD diffractometer
Absorption correction: multi-scan (*SADABS*; Bruker, 2009 ▶) *T*_min_ = 0.971, *T*_max_ = 0.983
21248 measured reflections
5516 independent reflections
3857 reflections with *I* > 2σ(*I*)
*R*_int_ = 0.058

#### Refinement

*R*[*F* ^2^ > 2σ(*F* ^2^)] = 0.051
*wR*(*F* ^2^) = 0.129
*S* = 1.03
5516 reflections
279 parameters
H atoms treated by a mixture of independent and constrained refinement
Δρ_max_ = 0.27 e Å^−3^
Δρ_min_ = −0.27 e Å^−3^

### 

Data collection: *APEX2* (Bruker, 2009 ▶); cell refinement: *SAINT* (Bruker, 2009 ▶); data reduction: *SAINT*; program(s) used to solve structure: *SHELXTL* (Sheldrick, 2008 ▶); program(s) used to refine structure: *SHELXTL*; molecular graphics: *SHELXTL*; software used to prepare material for publication: *SHELXTL* and *PLATON* (Spek, 2009 ▶).

## Supplementary Material

Crystal structure: contains datablock(s) global, I. DOI: 10.1107/S1600536811050239/hb6529sup1.cif

Structure factors: contains datablock(s) I. DOI: 10.1107/S1600536811050239/hb6529Isup2.hkl

Supplementary material file. DOI: 10.1107/S1600536811050239/hb6529Isup3.cml

Additional supplementary materials:  crystallographic information; 3D view; checkCIF report

## Figures and Tables

**Table 1:** Hydrogen-bond geometry (Å, °) *Cg*1 and *Cg*2 are the centroids of the C18–C23 and N3/N4/C15–C17 rings, respectively.

*D*—H⋯*A*	*D*—H	H⋯*A*	*D*⋯*A*	*D*—H⋯*A*
O1*W*—H1*W*1⋯N4^i^	0.86	2.13	2.9625 (18)	163
O1*W*—H2*W*1⋯O2^ii^	0.89	2.12	2.9465 (16)	154
N3—H1*N*3⋯O2^iii^	0.95 (2)	2.52 (3)	3.2162 (18)	130.4 (19)
N3—H1*N*3⋯N2^iii^	0.95 (2)	2.10 (3)	2.9927 (19)	155 (2)
N1—H1*N*1⋯O1*W*	0.94 (3)	2.06 (3)	2.9388 (18)	155 (2)
C14—H14*A*⋯O1*W*	0.95	2.54	3.2877 (18)	136
C16—H16*A*⋯N4^iv^	0.95	2.50	3.430 (2)	167
C21—H21*A*⋯O2^v^	0.95	2.53	3.318 (2)	140
C7—H7*A*⋯*Cg*2^vi^	0.95	2.80	3.6022 (18)	142
C22—H22*A*⋯*Cg*1^ii^	0.95	2.93	3.5274 (16)	122

Symmetry codes: (i) 
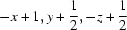
; (ii) 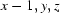; (iii) 
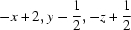
; (iv) 
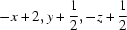
; (v) 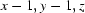; (vi) 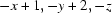.

**Fig. 1.**
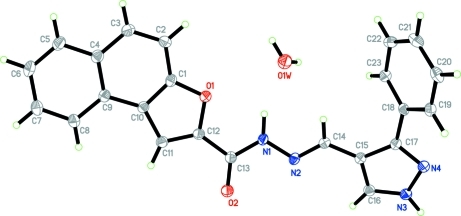
The molecular structure of the title compound, showing 50% probability displacement ellipsoids.

**Fig. 2.**
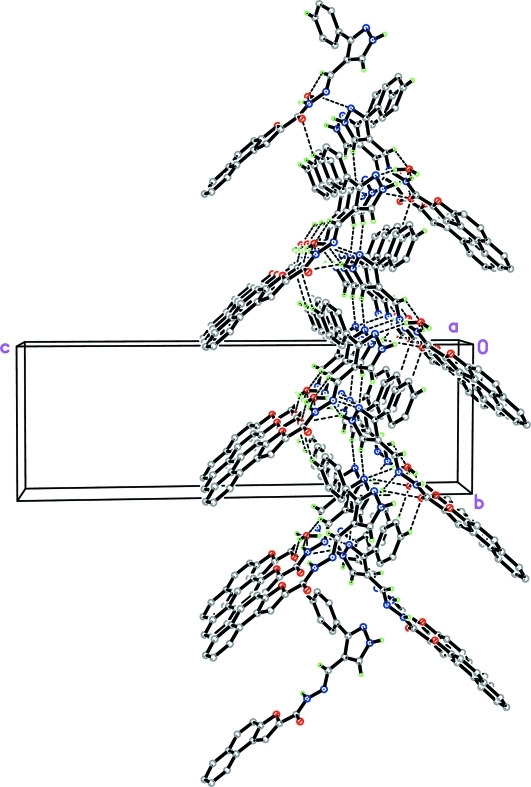
The crystal packing of the title compound, viewed along the a axis, showing the sheets parallel to the ab plane. H atoms not involved in the intermolecular interactions (dashed lines) have been omitted for clarity.

## supplementary crystallographic information

### Comment

Acidhydrazones and their condensation products possessing an azometine –NHN═ CH– proton constitute an important class of compounds for new drug development. In the past several years, numerous compounds with diverse structural features have been reported. Therefore, many researchers have synthesized these compounds as target structures and evaluated their biological activities. Hydrazides, hydrazones and their adducts have displayed diverse range of biological properties such as anti-viral (Jahagirdar *et al.*, 1990), anti-tuberculosis (Cavier *et al.*, 1965) and anti-inflammatory activities (Silva *et al.*, 2005; Eissa *et al.*, 2009). We have synthesized the title compound to study its crystal structure and evaluate its biological activities.

The title compound (Fig. 1) consists of one molecule of *N*'-[(*E*)-(3-phenyl-1*H*-pyrazol-4-yl)methylidene]naphtho [2,1-*b*]furan-2-carbohydrazide and a water molecule. The pyrazole ring (N3/N4/C15–C17) is approximately planar with a maximum deviation of 0.023 (1) Å at atom C17. This ring makes dihedral angles of 28.63 (6)° with the naphtho[2,1-*b*]furan ring system (O1/C1–C12; maximum deviation of 0.016 (1) Å at atom C10) and 46.44 (7)° with the benzene ring (C18–C23; maximum deviation of 0.012 (1) Å at atom C18). Bond lengths and angles are comparable to a related structure (Choi *et al.*, 2009).

In the crystal, (Fig. 2), O1W—H1W1···N4, O1W—H2W1···O2, N3—H1N3···O2, N3—H1N3···N2, N1—H1N1···O1W C14—H14A···O1W, C16—H16A···N4 and C21—H21A···O2 hydrogen bonds (Table 1) link the molecules to form sheets parallel to the *ab* plane. The crystal structure is further stabilized by C—H···π interactions (Table 1) involving the centroids of pyrazole (*Cg*1) and benzene (*Cg*2) rings.

### Experimental

A mixture of naphtho[2,1-*b*]furan-2-carbohydrazide (0.226 g, 0.001 mol) and 3-phenyl-1*H*-pyrazole-4-carbaldehyde (0.189 g, 0.0011 mol) was refluxed in ethanol for 4 h in the presence of a catalytic amount of acetic acid. The mixture was then cooled to room temperature and the resulting solid was filtered and dried to get the title compound. Yield: 0.28 g, 73.68%. *M.p.*: 524–526 K.

### Refinement

The O– and N-bound hydrogen atoms were located from the difference Fourier map. N-bound hydrogen atoms were refined freely and O-bound hydrogen atoms were fixed at their found positions with a riding model with *U*_iso_(H) = 1.2 *U*_eq_(O) [N–H= 0.94 (2) and 0.96 (2) Å; O–H = 0.8628 and 0.8895 Å]. The remaining hydrogen atoms were positioned geometrically and were refined with a riding model with *U*_iso_(H) = 1.2 *U*_eq_(C) [C–H = 0.95 Å].

### Crystal data

**Table:**

C_23_H_16_N_4_O_2_·H_2_O	*F*(000) = 832
*M**_r_* = 398.41	*D*_x_ = 1.399 Mg m^−^^3^
Monoclinic, *P*2_1_/*c*	Mo *K*α radiation, λ = 0.71073 Å
Hall symbol: -P 2ybc	Cell parameters from 3650 reflections
*a* = 7.1383 (1) Å	θ = 2.3–29.5°
*b* = 9.3928 (1) Å	µ = 0.10 mm^−^^1^
*c* = 28.4200 (4) Å	*T* = 100 K
β = 96.864 (1)°	Block, colourless
*V* = 1891.86 (4) Å^3^	0.31 × 0.25 × 0.18 mm
*Z* = 4	

### Data collection

**Table:**

Bruker SMART APEXII CCD diffractometer	5516 independent reflections
Radiation source: fine-focus sealed tube	3857 reflections with *I* > 2σ(*I*)
graphite	*R*_int_ = 0.058
φ and ω scans	θ_max_ = 30.1°, θ_min_ = 1.4°
Absorption correction: multi-scan (*SADABS*; Bruker, 2009)	*h* = −9→10
*T*_min_ = 0.971, *T*_max_ = 0.983	*k* = −13→13
21248 measured reflections	*l* = −39→40

### Refinement

**Table:**

Refinement on *F*^2^	Primary atom site location: structure-invariant direct methods
Least-squares matrix: full	Secondary atom site location: difference Fourier map
*R*[*F*^2^ > 2σ(*F*^2^)] = 0.051	Hydrogen site location: inferred from neighbouring sites
*wR*(*F*^2^) = 0.129	H atoms treated by a mixture of independent and constrained refinement
*S* = 1.03	*w* = 1/[σ^2^(*F*_o_^2^) + (0.0566*P*)^2^ + 0.4389*P*] where *P* = (*F*_o_^2^ + 2*F*_c_^2^)/3
5516 reflections	(Δ/σ)_max_ < 0.001
279 parameters	Δρ_max_ = 0.27 e Å^−^^3^
0 restraints	Δρ_min_ = −0.27 e Å^−^^3^

### Special details

**Table:**

Experimental. The crystal was placed in the cold stream of an Oxford Cryosystems Cobra open-flow nitrogen cryostat (Cosier & Glazer, 1986) operating at 100.0 (1) K.
Geometry. All e.s.d.'s (except the e.s.d. in the dihedral angle between two l.s. planes) are estimated using the full covariance matrix. The cell e.s.d.'s are taken into account individually in the estimation of e.s.d.'s in distances, angles and torsion angles; correlations between e.s.d.'s in cell parameters are only used when they are defined by crystal symmetry. An approximate (isotropic) treatment of cell e.s.d.'s is used for estimating e.s.d.'s involving l.s. planes.
Refinement. Refinement of *F*^2^ against ALL reflections. The weighted *R*-factor *wR* and goodness of fit *S* are based on *F*^2^, conventional *R*-factors *R* are based on *F*, with *F* set to zero for negative *F*^2^. The threshold expression of *F*^2^ > σ(*F*^2^) is used only for calculating *R*-factors(gt) *etc*. and is not relevant to the choice of reflections for refinement. *R*-factors based on *F*^2^ are statistically about twice as large as those based on *F*, and *R*- factors based on ALL data will be even larger.

### Fractional atomic coordinates and isotropic or equivalent isotropic displacement parameters (Å^2^)

**Table:**

	*x*	*y*	*z*	*U*_iso_*/*U*_eq_	
O1	0.43329 (14)	1.04167 (12)	0.06904 (4)	0.0190 (2)	
O2	0.91817 (15)	1.01592 (13)	0.11647 (4)	0.0251 (3)	
N1	0.65264 (19)	0.90451 (14)	0.13601 (4)	0.0177 (3)	
N2	0.75457 (18)	0.82808 (14)	0.17263 (4)	0.0177 (3)	
N3	0.93853 (18)	0.49668 (15)	0.27204 (5)	0.0196 (3)	
N4	0.78932 (17)	0.40748 (14)	0.26062 (5)	0.0196 (3)	
C1	0.3524 (2)	1.12767 (17)	0.03335 (5)	0.0183 (3)	
C2	0.1589 (2)	1.13360 (19)	0.01844 (6)	0.0232 (3)	
H2A	0.0711	1.0769	0.0327	0.028*	
C3	0.1027 (2)	1.22541 (19)	−0.01779 (6)	0.0245 (4)	
H3A	−0.0275	1.2308	−0.0294	0.029*	
C4	0.2337 (2)	1.31378 (18)	−0.03878 (6)	0.0212 (3)	
C5	0.1705 (2)	1.41189 (19)	−0.07502 (6)	0.0242 (4)	
H5A	0.0398	1.4178	−0.0861	0.029*	
C6	0.2963 (2)	1.49881 (18)	−0.09438 (6)	0.0248 (4)	
H6A	0.2514	1.5661	−0.1180	0.030*	
C7	0.4909 (2)	1.48864 (18)	−0.07936 (6)	0.0238 (3)	
H7A	0.5772	1.5474	−0.0934	0.029*	
C8	0.5563 (2)	1.39364 (17)	−0.04433 (6)	0.0210 (3)	
H8A	0.6878	1.3874	−0.0343	0.025*	
C9	0.4303 (2)	1.30561 (17)	−0.02320 (5)	0.0185 (3)	
C10	0.4875 (2)	1.20827 (16)	0.01465 (5)	0.0170 (3)	
C11	0.6639 (2)	1.17157 (17)	0.04153 (5)	0.0188 (3)	
H11A	0.7846	1.2095	0.0377	0.023*	
C12	0.6241 (2)	1.07205 (17)	0.07342 (5)	0.0175 (3)	
C13	0.7456 (2)	0.99555 (17)	0.11028 (5)	0.0184 (3)	
C14	0.6704 (2)	0.71368 (16)	0.18341 (5)	0.0167 (3)	
H14A	0.5497	0.6931	0.1669	0.020*	
C15	0.7500 (2)	0.61507 (16)	0.21930 (5)	0.0166 (3)	
C16	0.9210 (2)	0.61994 (17)	0.24849 (5)	0.0185 (3)	
H16A	1.0087	0.6965	0.2512	0.022*	
C17	0.6738 (2)	0.47933 (16)	0.22852 (5)	0.0168 (3)	
C18	0.4979 (2)	0.41425 (16)	0.20556 (5)	0.0169 (3)	
C19	0.4996 (2)	0.27495 (17)	0.18791 (5)	0.0197 (3)	
H19A	0.6122	0.2203	0.1927	0.024*	
C20	0.3376 (2)	0.21643 (18)	0.16353 (6)	0.0229 (3)	
H20A	0.3396	0.1219	0.1517	0.027*	
C21	0.1728 (2)	0.29580 (19)	0.15644 (6)	0.0236 (4)	
H21A	0.0629	0.2564	0.1391	0.028*	
C22	0.1685 (2)	0.43245 (18)	0.17454 (5)	0.0209 (3)	
H22A	0.0550	0.4862	0.1700	0.025*	
C23	0.3301 (2)	0.49127 (17)	0.19929 (5)	0.0187 (3)	
H23A	0.3259	0.5846	0.2120	0.022*	
O1W	0.25339 (16)	0.83476 (13)	0.13979 (4)	0.0270 (3)	
H1W1	0.2518	0.8380	0.1701	0.041*	
H2W1	0.1460	0.8694	0.1251	0.041*	
H1N3	1.036 (3)	0.467 (3)	0.2959 (9)	0.064 (8)*	
H1N1	0.523 (4)	0.888 (3)	0.1276 (9)	0.061 (7)*	

### Atomic displacement parameters (Å^2^)

**Table:**

	*U*^11^	*U*^22^	*U*^33^	*U*^12^	*U*^13^	*U*^23^
O1	0.0172 (5)	0.0208 (6)	0.0183 (5)	−0.0003 (4)	−0.0002 (4)	0.0029 (4)
O2	0.0196 (6)	0.0262 (6)	0.0277 (6)	−0.0049 (5)	−0.0040 (4)	0.0068 (5)
N1	0.0180 (6)	0.0176 (6)	0.0168 (6)	−0.0007 (5)	−0.0016 (5)	0.0038 (5)
N2	0.0193 (6)	0.0173 (6)	0.0158 (6)	0.0009 (5)	−0.0011 (5)	0.0011 (5)
N3	0.0163 (6)	0.0231 (7)	0.0185 (6)	−0.0006 (5)	−0.0014 (5)	0.0028 (6)
N4	0.0175 (6)	0.0204 (7)	0.0203 (7)	0.0000 (5)	0.0005 (5)	0.0027 (5)
C1	0.0208 (7)	0.0189 (7)	0.0150 (7)	0.0015 (6)	0.0013 (6)	0.0009 (6)
C2	0.0181 (8)	0.0287 (9)	0.0229 (8)	−0.0027 (7)	0.0024 (6)	0.0043 (7)
C3	0.0189 (8)	0.0305 (9)	0.0238 (8)	0.0017 (7)	0.0017 (6)	0.0034 (7)
C4	0.0222 (8)	0.0233 (8)	0.0178 (7)	0.0020 (7)	0.0017 (6)	0.0003 (6)
C5	0.0240 (8)	0.0289 (9)	0.0191 (8)	0.0053 (7)	0.0004 (6)	0.0024 (7)
C6	0.0334 (9)	0.0234 (8)	0.0176 (8)	0.0068 (7)	0.0025 (6)	0.0032 (7)
C7	0.0299 (9)	0.0229 (8)	0.0192 (8)	−0.0009 (7)	0.0055 (6)	0.0010 (7)
C8	0.0238 (8)	0.0203 (8)	0.0193 (8)	0.0000 (6)	0.0047 (6)	0.0010 (6)
C9	0.0207 (8)	0.0195 (8)	0.0151 (7)	0.0011 (6)	0.0024 (6)	−0.0006 (6)
C10	0.0175 (7)	0.0181 (7)	0.0152 (7)	0.0011 (6)	0.0013 (5)	−0.0003 (6)
C11	0.0173 (7)	0.0194 (8)	0.0195 (7)	0.0003 (6)	0.0011 (6)	0.0002 (6)
C12	0.0161 (7)	0.0181 (7)	0.0179 (7)	−0.0015 (6)	0.0002 (5)	−0.0006 (6)
C13	0.0195 (7)	0.0176 (7)	0.0176 (7)	−0.0015 (6)	−0.0004 (6)	0.0001 (6)
C14	0.0166 (7)	0.0170 (7)	0.0164 (7)	0.0011 (6)	0.0009 (5)	−0.0014 (6)
C15	0.0177 (7)	0.0169 (7)	0.0153 (7)	0.0015 (6)	0.0022 (5)	−0.0008 (6)
C16	0.0180 (7)	0.0202 (8)	0.0173 (7)	−0.0004 (6)	0.0028 (6)	0.0008 (6)
C17	0.0182 (7)	0.0173 (7)	0.0150 (7)	0.0024 (6)	0.0029 (5)	−0.0001 (6)
C18	0.0193 (7)	0.0173 (7)	0.0141 (7)	−0.0024 (6)	0.0020 (5)	0.0027 (6)
C19	0.0223 (8)	0.0179 (8)	0.0194 (8)	0.0008 (6)	0.0039 (6)	0.0021 (6)
C20	0.0292 (9)	0.0193 (8)	0.0213 (8)	−0.0066 (7)	0.0075 (6)	−0.0027 (6)
C21	0.0230 (8)	0.0291 (9)	0.0190 (8)	−0.0091 (7)	0.0035 (6)	−0.0016 (7)
C22	0.0162 (7)	0.0268 (9)	0.0195 (8)	−0.0004 (6)	0.0015 (6)	0.0003 (7)
C23	0.0191 (7)	0.0185 (8)	0.0186 (7)	0.0004 (6)	0.0021 (6)	−0.0007 (6)
O1W	0.0273 (6)	0.0292 (7)	0.0257 (6)	0.0054 (5)	0.0076 (5)	0.0018 (5)

### Geometric parameters (Å, °)

**Table:**

O1—C1	1.3692 (18)	C8—H8A	0.9500
O1—C12	1.3826 (17)	C9—C10	1.433 (2)
O2—C13	1.2383 (18)	C10—C11	1.435 (2)
N1—C13	1.3499 (19)	C11—C12	1.356 (2)
N1—N2	1.3954 (17)	C11—H11A	0.9500
N1—H1N1	0.94 (2)	C12—C13	1.466 (2)
N2—C14	1.2860 (19)	C14—C15	1.443 (2)
N3—C16	1.336 (2)	C14—H14A	0.9500
N3—N4	1.3636 (18)	C15—C16	1.392 (2)
N3—H1N3	0.96 (2)	C15—C17	1.423 (2)
N4—C17	1.3372 (19)	C16—H16A	0.9500
C1—C10	1.381 (2)	C17—C18	1.476 (2)
C1—C2	1.397 (2)	C18—C23	1.393 (2)
C2—C3	1.366 (2)	C18—C19	1.402 (2)
C2—H2A	0.9500	C19—C20	1.389 (2)
C3—C4	1.433 (2)	C19—H19A	0.9500
C3—H3A	0.9500	C20—C21	1.387 (2)
C4—C5	1.415 (2)	C20—H20A	0.9500
C4—C9	1.422 (2)	C21—C22	1.384 (2)
C5—C6	1.376 (2)	C21—H21A	0.9500
C5—H5A	0.9500	C22—C23	1.392 (2)
C6—C7	1.407 (2)	C22—H22A	0.9500
C6—H6A	0.9500	C23—H23A	0.9500
C7—C8	1.376 (2)	O1W—H1W1	0.8628
C7—H7A	0.9500	O1W—H2W1	0.8895
C8—C9	1.408 (2)		
			
C1—O1—C12	105.57 (12)	C12—C11—H11A	126.8
C13—N1—N2	118.93 (13)	C10—C11—H11A	126.8
C13—N1—H1N1	119.8 (15)	C11—C12—O1	111.43 (13)
N2—N1—H1N1	121.1 (15)	C11—C12—C13	131.48 (14)
C14—N2—N1	113.00 (12)	O1—C12—C13	117.09 (13)
C16—N3—N4	112.94 (13)	O2—C13—N1	124.46 (14)
C16—N3—H1N3	128.8 (15)	O2—C13—C12	121.30 (14)
N4—N3—H1N3	118.2 (15)	N1—C13—C12	114.24 (13)
C17—N4—N3	104.65 (13)	N2—C14—C15	123.27 (14)
O1—C1—C10	110.89 (13)	N2—C14—H14A	118.4
O1—C1—C2	124.22 (14)	C15—C14—H14A	118.4
C10—C1—C2	124.89 (14)	C16—C15—C17	104.31 (13)
C3—C2—C1	116.37 (15)	C16—C15—C14	130.05 (14)
C3—C2—H2A	121.8	C17—C15—C14	125.31 (14)
C1—C2—H2A	121.8	N3—C16—C15	107.04 (14)
C2—C3—C4	122.18 (15)	N3—C16—H16A	126.5
C2—C3—H3A	118.9	C15—C16—H16A	126.5
C4—C3—H3A	118.9	N4—C17—C15	111.07 (13)
C5—C4—C9	118.60 (15)	N4—C17—C18	121.01 (14)
C5—C4—C3	120.88 (15)	C15—C17—C18	127.84 (13)
C9—C4—C3	120.51 (14)	C23—C18—C19	118.89 (14)
C6—C5—C4	120.73 (15)	C23—C18—C17	120.96 (14)
C6—C5—H5A	119.6	C19—C18—C17	120.10 (14)
C4—C5—H5A	119.6	C20—C19—C18	120.38 (15)
C5—C6—C7	120.41 (15)	C20—C19—H19A	119.8
C5—C6—H6A	119.8	C18—C19—H19A	119.8
C7—C6—H6A	119.8	C21—C20—C19	120.06 (15)
C8—C7—C6	120.04 (15)	C21—C20—H20A	120.0
C8—C7—H7A	120.0	C19—C20—H20A	120.0
C6—C7—H7A	120.0	C22—C21—C20	120.04 (15)
C7—C8—C9	120.71 (15)	C22—C21—H21A	120.0
C7—C8—H8A	119.6	C20—C21—H21A	120.0
C9—C8—H8A	119.6	C21—C22—C23	120.13 (15)
C8—C9—C4	119.48 (14)	C21—C22—H22A	119.9
C8—C9—C10	123.75 (14)	C23—C22—H22A	119.9
C4—C9—C10	116.75 (14)	C22—C23—C18	120.45 (15)
C1—C10—C9	119.27 (14)	C22—C23—H23A	119.8
C1—C10—C11	105.79 (13)	C18—C23—H23A	119.8
C9—C10—C11	134.91 (14)	H1W1—O1W—H2W1	110.0
C12—C11—C10	106.30 (13)		
			
C13—N1—N2—C14	−157.33 (14)	C1—O1—C12—C11	−0.81 (17)
C16—N3—N4—C17	−0.15 (17)	C1—O1—C12—C13	178.90 (13)
C12—O1—C1—C10	1.37 (17)	N2—N1—C13—O2	0.8 (2)
C12—O1—C1—C2	−177.96 (15)	N2—N1—C13—C12	−178.86 (13)
O1—C1—C2—C3	−179.88 (15)	C11—C12—C13—O2	−0.6 (3)
C10—C1—C2—C3	0.9 (3)	O1—C12—C13—O2	179.70 (14)
C1—C2—C3—C4	−1.5 (3)	C11—C12—C13—N1	178.97 (16)
C2—C3—C4—C5	−177.51 (16)	O1—C12—C13—N1	−0.7 (2)
C2—C3—C4—C9	1.8 (3)	N1—N2—C14—C15	178.31 (13)
C9—C4—C5—C6	−0.8 (2)	N2—C14—C15—C16	0.5 (3)
C3—C4—C5—C6	178.56 (16)	N2—C14—C15—C17	−171.90 (14)
C4—C5—C6—C7	1.8 (3)	N4—N3—C16—C15	0.06 (17)
C5—C6—C7—C8	−1.5 (3)	C17—C15—C16—N3	0.06 (16)
C6—C7—C8—C9	0.1 (2)	C14—C15—C16—N3	−173.53 (15)
C7—C8—C9—C4	0.9 (2)	N3—N4—C17—C15	0.18 (16)
C7—C8—C9—C10	−177.53 (15)	N3—N4—C17—C18	177.03 (13)
C5—C4—C9—C8	−0.6 (2)	C16—C15—C17—N4	−0.15 (17)
C3—C4—C9—C8	−179.93 (15)	C14—C15—C17—N4	173.83 (14)
C5—C4—C9—C10	177.97 (14)	C16—C15—C17—C18	−176.73 (14)
C3—C4—C9—C10	−1.4 (2)	C14—C15—C17—C18	−2.8 (2)
O1—C1—C10—C9	−179.87 (13)	N4—C17—C18—C23	136.78 (15)
C2—C1—C10—C9	−0.5 (2)	C15—C17—C18—C23	−46.9 (2)
O1—C1—C10—C11	−1.39 (17)	N4—C17—C18—C19	−45.9 (2)
C2—C1—C10—C11	177.93 (15)	C15—C17—C18—C19	130.41 (16)
C8—C9—C10—C1	179.24 (15)	C23—C18—C19—C20	1.6 (2)
C4—C9—C10—C1	0.8 (2)	C17—C18—C19—C20	−175.76 (14)
C8—C9—C10—C11	1.3 (3)	C18—C19—C20—C21	0.1 (2)
C4—C9—C10—C11	−177.16 (16)	C19—C20—C21—C22	−1.4 (2)
C1—C10—C11—C12	0.84 (17)	C20—C21—C22—C23	0.9 (2)
C9—C10—C11—C12	178.97 (17)	C21—C22—C23—C18	0.8 (2)
C10—C11—C12—O1	−0.02 (18)	C19—C18—C23—C22	−2.1 (2)
C10—C11—C12—C13	−179.69 (16)	C17—C18—C23—C22	175.28 (14)

### Hydrogen-bond geometry (Å, °)

**Table:**

Cg1 and Cg2 are the centroids of the C18–C23 and N3/N4/C17/C15/C16 rings, respectively.

**Table:**

*D*—H···*A*	*D*—H	H···*A*	*D*···*A*	*D*—H···*A*
O1W—H1W1···N4^i^	0.86	2.13	2.9625 (18)	163
O1W—H2W1···O2^ii^	0.89	2.12	2.9465 (16)	154
N3—H1N3···O2^iii^	0.95 (2)	2.52 (3)	3.2162 (18)	130.4 (19)
N3—H1N3···N2^iii^	0.95 (2)	2.10 (3)	2.9927 (19)	155 (2)
N1—H1N1···O1W	0.94 (3)	2.06 (3)	2.9388 (18)	155 (2)
C14—H14A···O1W	0.95	2.54	3.2877 (18)	136
C16—H16A···N4^iv^	0.95	2.50	3.430 (2)	167
C21—H21A···O2^v^	0.95	2.53	3.318 (2)	140
C7—H7A···Cg2^vi^	0.95	2.80	3.6022 (18)	142
C22—H22A···Cg1^ii^	0.95	2.93	3.5274 (16)	122

Symmetry codes: (i) −*x*+1, *y*+1/2, −*z*+1/2; (ii) *x*−1, *y*, *z*; (iii) −*x*+2, *y*−1/2, −*z*+1/2; (iv) −*x*+2, *y*+1/2, −*z*+1/2; (v) *x*−1, *y*−1, *z*; (vi) −*x*+1, −*y*+2, −*z*.
